# Tailored Therapeutic Approach in a Patient With Diffuse Large B‐Cell Lymphoma With Meningeal Infiltration, Concurrent Classic Hodgkin Lymphoma, and Smoldering Multiple Myeloma: A Case Report

**DOI:** 10.1002/cnr2.70102

**Published:** 2025-04-15

**Authors:** Giusy Ceparano, Daniele Lorenzini, Maurilio Ponzoni, Giorgia Levati, Davide Epifania, Vincenzo Marasco

**Affiliations:** ^1^ Department of Hematology and Stem Cell Transplantation Fondazione IRCCS Istituto Nazionale dei Tumori Milan Italy; ^2^ Hematology University of Milan Milan Italy; ^3^ Department of Pathology Fondazione IRCCS Istituto Nazionale dei Tumori Milan Italy; ^4^ Pathology Unit IRCCS San Raffaele Scientific Institute Milan Italy

**Keywords:** concurrent neoplasms, diffuse large B‐cell lymphoma, Hodgkin lymphoma, multiple myeloma, tailored therapeutic approach

## Abstract

**Background:**

Coexisting primary hematologic malignancies in untreated multiple myeloma (MM) are rare.

**Case:**

A 69‐year‐old man with smoldering multiple myeloma (sMM) presented with lymphadenopathies and an intracranial mass. He was diagnosed with diffuse large B‐cell lymphoma (DLBCL), classic Hodgkin lymphoma (cHL), and sMM. The patient received R‐CHOP and R‐ICE, followed by autologous stem cell transplantation, achieving complete remission of DLBCL and cHL, and very good partial remission of MM.

**Conclusion:**

This case illustrates the complexity of managing multiple malignancies and the importance of a tailored therapeutic approach.

## 1 Introduction

B‐cell neoplasms are highly heterogeneous diseases and differentiate based on specific genetic alterations that can occur at various stages of the ontogenesis of B lymphocytes. The simultaneous presence of two or more of these pathologies can be challenging regarding the clinical and therapeutic management of the patient, as treatments can be extremely diverse, and a “one size fits all” approach may not always be applicable.

While multiple myeloma (MM) is known to be associated with secondary hematologic malignancies, these typically arise later in the disease course as a consequence of prior treatments, including immunomodulatory therapies, high‐dose chemotherapy, or autologous stem cell transplantation [1]. The emergence of another primary hematologic malignancy in an untreated MM patient is exceedingly rare, with only a few documented cases [2]. Concurrent diagnoses of diffuse large B‐cell lymphoma (DLBCL) and classic Hodgkin lymphoma (cHL) have been previously reported, but not in the context of smoldering multiple myeloma (SMM), which remains asymptomatic and typically does not require immediate treatment [3, 4].

Here, we present a unique case of a patient with smoldering multiple myeloma who developed two concurrent lymphoproliferative disorders—cHL and DLBCL with central nervous system (CNS) involvement—at the National Cancer Institute in Milan, Italy, in October 2022. To our knowledge, this is the first reported case of its kind, highlighting the diagnostic and therapeutic challenges of managing three simultaneous hematologic malignancies. This case underscores the importance of a tailored therapeutic approach in the management of such complex presentations.

### 1.1 Case Report

In December 2004, a previously healthy 51‐year‐old man was diagnosed with IgG‐k monoclonal gammopathy of undetermined significance at National Cancer Institute in Milan. At the time of diagnosis, his monoclonal spike was 300 mg/dL. He continued annual follow‐up with urine and blood exams, developing a slow increase in serum M‐protein over the years.

Seventeen years later, the patient started to complain of abdominal pain and fatigue and a rapidly growing mass on the right parietal side of the cranial vault. Blood exams were unremarkable except for an increase in serum IgG‐k M‐protein up to 1540 mg/dL and serum kappa freelite chain of 24 mg/L with kappa‐lambda ratio of 1.69. Spine magnetic resonance imaging (MRI) and total‐body skeletal computed tomography (CT) excluded MM bone lesions.

**FIGURE 1:**
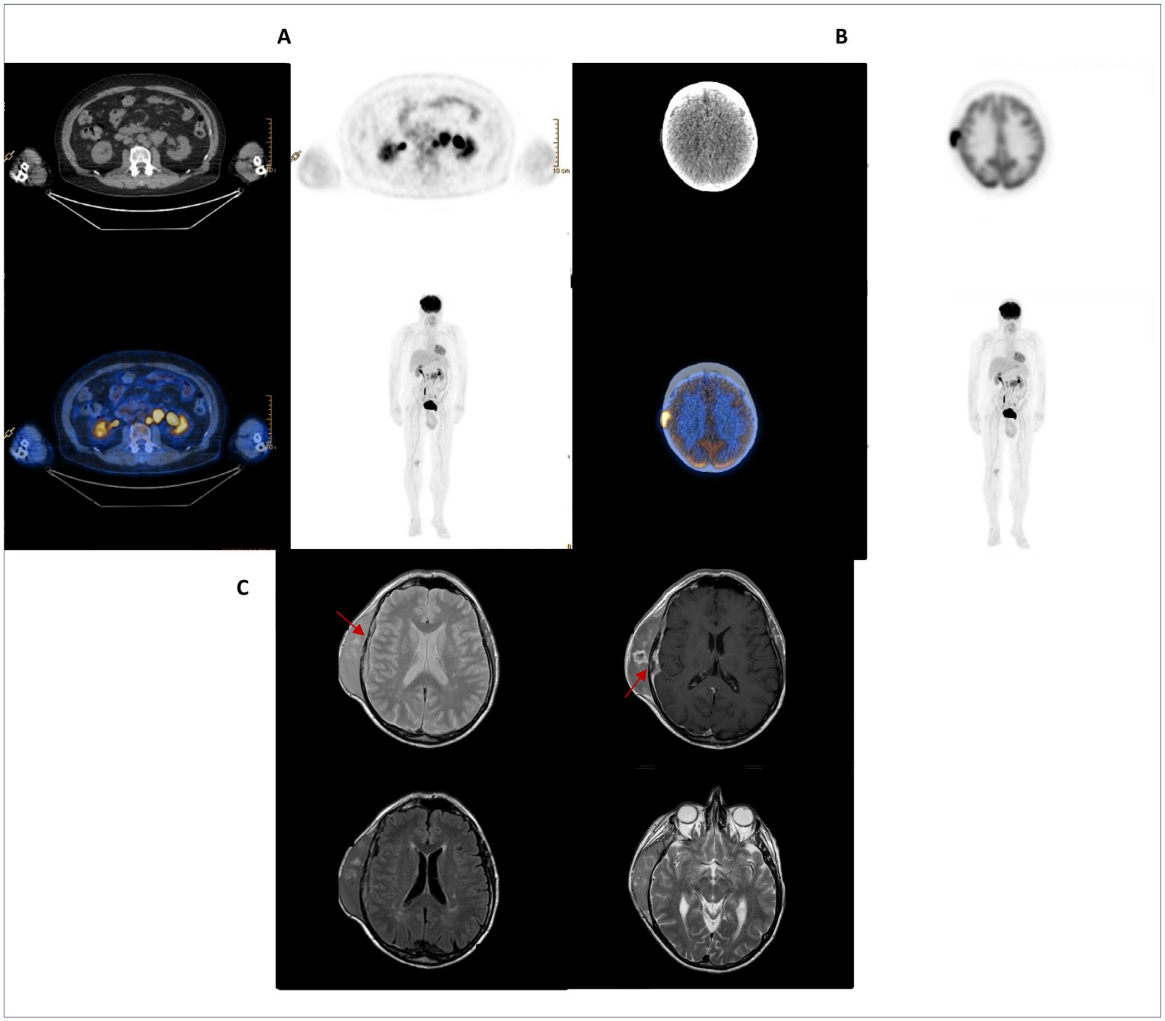
FDG‐PET evaluation (A and B) and brain MRI scan showing meningeal infiltration (C) at diagnosis.

A contrast CT scan revealed the presence of para‐aortic lymph nodes measuring 39 × 38mm; 18‐fluorodeoxy‐glucose‐positron emission tomography (18FDG‐PET) detected multiple FDG‐avid abdominal lymphadenopathies (SUVmax 10) (Figure 1A) and a large right parietal mass with extremely intense FDG‐uptake (SUVmax 28) (Figure 1B); brain MRI confirmed the presence of a mass involving the subcutaneous tissue and the right frontal, parietal, and temporal bones, with contiguous meningeal infiltration [Figure 1C].

**FIGURE 2:**
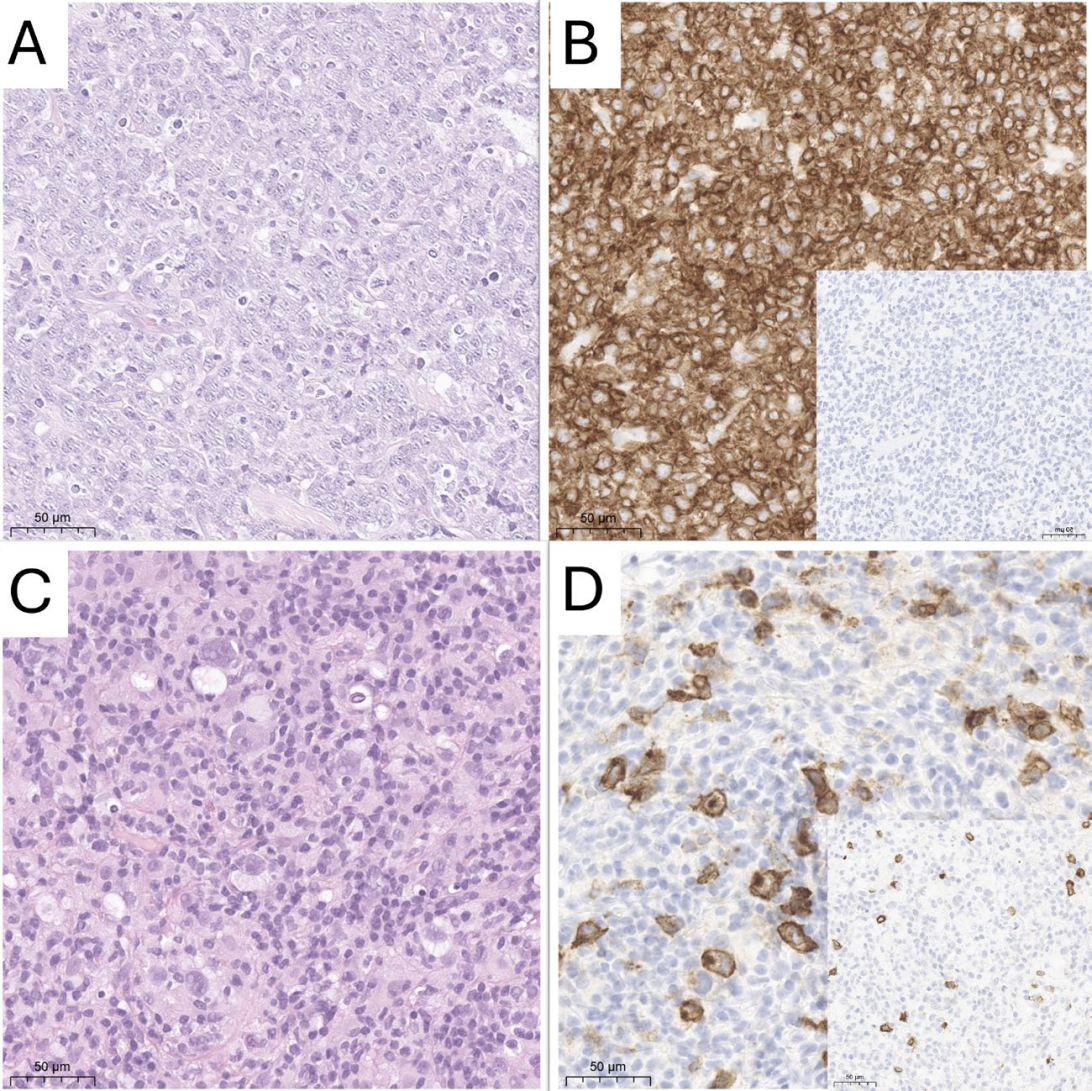
Right parietal mass biopsy showing a diffuse infiltration by medium to large lymphocytes in dense sheet (A); immunohistochemical staining showing strong CD79a expression by lymphomatous cells (B), which are negative for CD30 (insert); core biopsies of abdominal lymphadenopathy showing a mixed infiltration of small lymphocytes, histiocytes and granulocytes, with few interspersed Hodgkin and Reed‐Sternberg cells (C); immunohistochemical staining showing CD30 expression in Hodgkin and Reed‐Sternberg cells (D), which are negative for CD79a (insert).

Suspecting a lymphoproliferative disorder, a CT‐guided core biopsy of the retroperitoneal mass was undertaken. The histological analysis disclosed the presence of scattered large cells with Hodgkin and Reed‐Sternberg (HRS) morphology, interspersed in a mixture of small lymphocytes, plasma cells, histiocytes and granulocytes; immunohistochemical characterization of HRS cells showed positivity for CD30, MUM1 and PAX5, without expression of CD15 or other pan‐B or plasma cell markers (CD19, CD20, CD79a, CD45, CD138, kappa and lambda light chain); a diagnosis of classic Hodgkin lymphoma was made (Figure 2C,D). In situ hybridization for Epstein–Barr virus‐encoded small RNAs (EBER) turned out negative.

Considering the different FDG‐PET uptake of the two masses, we decided to conduct a stereotactic biopsy of the parietal mass as well. Surprisingly, histological analysis revealed a disparate population of large B cells in large confluent sheets with strong and uniform expression of pan‐B cell markers (CD20, CD79a), without expression of CD30, CD15 or plasma cell markers (CD38, CD138) and a non‐germinal center phenotype (CD10−, BCL6+, MUM1+) (Figure 2A,B). Fluorescent in situ ibridization excluded *BCL2*, *BCL6* or *MYC* rearrangement; in situ ibridization for EBV/EBER turned out negative too. Hence, a diagnosis of diffuse large B‐cell lymphoma, non‐GCB type, was defined.

An attempt to compare immunoglobulin rearrangement in both lymphomas through direct sequencing was performed; as expected, however, while DLBCL showed a monoclonal IGK rearrangement, cHL resulted in a polyclonal profile in all regions analyzed. The potential genetic connection between the DLBCL and MM clones could not be investigated due to insufficient material.

Bone marrow biopsy was negative for lymphomatous infiltration; however, there was a plasma cell infiltration with monotypic kappa light chain restriction corresponding to 10% of all nucleated cells, consistent with a diagnosis of MM. In conclusion, the diagnostic work‐up for multiple myeloma ruled out the presence of SLiM‐CRAB criteria required to initiate treatment.

Considering the simultaneous occurrence of stage IIA cHL and stage IV DLBCL with contiguous CNS involvement, we decided to start the patient on chemo‐immunotherapy with 3 cycles of R‐CHOP followed by 3 cycles of R‐ICE, with associated lumbar punctures for CNS prophylaxis. Cerebrospinal fluid (CSF) flow cytometry and cytology were consistently negative for disease infiltration.

After 2 cycles of therapy, the patient achieved a partial remission.

Brain MRI, CT scan and FDG PET performed after 6 cycles confirmed a complete volumetric and metabolic response. Blood exams at the end of the treatment showed a reduction of IgG kappa M‐spike to 100 mg/dL, consistent with a very good partial response of MM.

The patient underwent stem cell mobilization after the fifth cycle of therapy with a collection of 6 × 10^4^ per kilogram peripheral blood CD34+ cells. He received autologous stem cell transplant after the sixth cycle. Considering the disease infiltration at the CNS level, we chose a conditioning regimen consisting of carmustine 300 mg/mq on day −6 and a total dose of 14 mg/kg of thiotepa administered in 4 doses on days −5 and −4. Post‐transplant complications included *C. difficile* infection, febrile neutropenia, and grade 2 mucositis.

Disease evaluation after transplant confirmed a complete metabolic response of HL and DLBCL, and a very good partial response of MM. The last follow‐up in September 2024 indicated that the patient is in good condition with a preserved quality of life and with maintained responses (Table 1).

**TABLE 1:** Timeline of major events in the patient's clinical history.

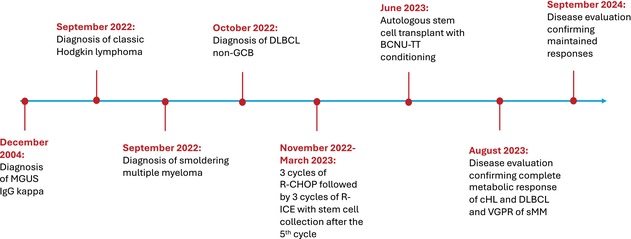

## 2 Discussion

The coexistence of two different neoplasms at the same time is a rare phenomenon.

Our case is particularly interesting as it describes a patient with the simultaneous occurrence of three different hematologic neoplasms: cHL, DLBCL with meningeal involvement, and SMM.

Smoldering Multiple Myeloma is a condition that does not require immediate treatment but a close follow‐up to detect evolution into symptomatic multiple myeloma. Some clinical trials evaluated the benefit of early treatment in high‐risk SMM, defined by the ‘2‐20‐20’ rule, but so far no specific treatment is recommended [3, 4]. In our patient, SMM could be stratified as low risk according to MAYO score, being serum M‐protein less than 2 g/dL, free‐light chain ratio less than 20, and bone marrow plasma cells less than 20%.

Diffuse large B‐cell lymphoma and cHL are aggressive B‐cell lymphoproliferative disorders with different incidence and clinical presentation.

Classic Hodgkin lymphoma affects primarily young people, with a second peak of incidence in people aged 55 years or older. The majority of cHL patients present with early‐stage disease and are cured in virtually > 80% of cases with combination chemotherapy followed by involved‐field irradiation; however, elderly patients can experience a worse outcome due to inferior response rates to standard treatment and comorbidities [5].

Diffuse large B‐cell lymphoma is the most common type of non‐Hodgkin lymphoma, presenting often in the advanced stage and as a rapidly growing mass. Standard treatment for DLBCL consists of chemo‐immunotherapy regimens such as R‐CHOP or R‐DAEPOCH that allow long‐term remissions in about 55%–60% of patients [6].

Sometimes systemic DLBCL can also be localized at the CNS level as Secondary Central Nervous System (CNS) Lymphoma (SCNSL). Tumor cells can spread to the central nervous system through hematogenous dissemination or by contiguity. Most involved sites include the brain parenchyma, meninges, cranial nerves, eyes and spinal cord.

The standard chemotherapy agents used for the treatment of systemic DLBCL lack efficacy in primary or secondary CNS lymphoma, as a result of poor penetration of the blood–brain barrier (BBB). The use of anti‐neoplastic agents with elevated BBB penetrance such as ifosfamide, etoposide, methotrexate and cytarabine is associated with higher response rates and progression free survival (PFS) and is currently the standard induction treatment followed by high‐dose chemotherapy (HDC) and autologous hematopoietic stem cell transplantation (ASCT) as a consolidation strategy for eligible patients [7, 8].

Due to meningeal infiltration of the parietal mass, even though negative CSF analysis, we decided to treat the patient as being affected by SCNSL. Hence, an induction strategy with chemotherapy agents of consolidated efficacy in cHL and DLBCL as well as elevated CNS penetrance was administered successfully, achieving a complete metabolic response. Considering the absence of significant comorbidity, we decided to consolidate the response with ASCT, using carmustine and thiotepa as a conditioning regimen due to high BBB penetrance [7, 8, 9], with dose reduction for age.

There is evidence of the existence of a clonal relationship between different hematologic neoplasms occurring simultaneously in the same patient. Previous case reports demonstrated the derivation from the same B‐cell lineage of concurrent B‐cell lymphoproliferative disorders using a direct sequencing method of CDR2, CDR3, and VH3 sequences of immunoglobulin genes [10]. To address the same question in our patient, we analyzed the *IGH* and *IGK* rearrangement of DLBCL and cHL cells, detecting a monoclonal rearrangement for DLBCL cells but polyclonal rearrangement for cHL. This is reasonably due to the characteristic reactive cellular background of HL and the relatively few Hodgkin and Reed‐Sternberg cells that invalidate the analysis of the immunoglobulin rearrangement.

In conclusion, to the best of our knowledge, this represents the first case report documenting the concomitant occurrence of three distinct hematologic neoplasms. We contend that our case warrants attention given the clinical complexity of managing three different diseases concurrently with an effective and safe customized treatment approach.

## Author Contributions

G.C. and V.M. wrote the manuscript; G.L. and D.E. reviewed the manuscript; D.L. and M.P. performed the histological examinations, provided iconographic materials, interpreted these data, and integrated them in the manuscript.

## Consent

The patient provided his written informed consent to participate in this study.

## Conflicts of Interest

The authors declare no conflicts of interest.

## Data Availability

The original contributions presented in the study are included in the article/Supporting Information. Further inquiries can be directed to the corresponding author.
